# Risk factors for COPD exacerbations in inhaled medication users: the COPDGene study biannual longitudinal follow-up prospective cohort

**DOI:** 10.1186/s12890-016-0191-7

**Published:** 2016-02-10

**Authors:** Robert Busch, MeiLan K. Han, Russell P. Bowler, Mark T. Dransfield, J Michael Wells, Elizabeth A. Regan, Craig P. Hersh, the COPDGene Investigators

**Affiliations:** Channing Division of Network Medicine, Brigham and Women’s Hospital, Harvard Medical School, 181 Longwood Avenue, Boston, MA 02115 USA; Division of Pulmonary and Critical Care Medicine, University of Michigan, Ann Arbor, MI USA; Department of Medicine, National Jewish Health, Denver, CO USA; Division of Pulmonary, Allergy, and Critical Care Medicine, University of Alabama at Birmingham, Birmingham, AL USA

**Keywords:** Chronic obstructive pulmonary disease, COPD exacerbation, Inhaled medications, Prospective cohort study, Long-acting beta-agonist, Inhaled corticosteroid, Tiotropium, Adrenergic beta-agonists

## Abstract

**Background:**

Despite inhaled medications that decrease exacerbation risk, some COPD patients experience frequent exacerbations. We determined prospective risk factors for exacerbations among subjects in the COPDGene Study taking inhaled medications.

**Methods:**

2113 COPD subjects were categorized into four medication use patterns: triple therapy with tiotropium (TIO) plus long-acting beta-agonist/inhaled-corticosteroid (ICS ± LABA), tiotropium alone, ICS ± LABA, and short-acting bronchodilators. Self-reported exacerbations were recorded in telephone and web-based longitudinal follow-up surveys. Associations with exacerbations were determined within each medication group using four separate logistic regression models. A head-to-head analysis compared exacerbation risk among subjects using tiotropium vs. ICS ± LABA.

**Results:**

In separate logistic regression models, the presence of gastroesophageal reflux, female gender, and higher scores on the St. George’s Respiratory Questionnaire were significant predictors of exacerbator status within multiple medication groups (reflux: OR 1.62–2.75; female gender: OR 1.53 - OR 1.90; SGRQ: OR 1.02–1.03). Subjects taking either ICS ± LABA or tiotropium had similar baseline characteristics, allowing comparison between these two groups. In the head-to-head comparison, tiotropium users showed a trend towards lower rates of exacerbations (OR = 0.69 [95 % CI 0.45, 1.06], *p* = 0.09) compared with ICS ± LABA users, especially in subjects without comorbid asthma (OR = 0.56 [95 % CI 0.31, 1.00], *p* = 0.05).

**Conclusions:**

Each common COPD medication usage group showed unique risk factor patterns associated with increased risk of exacerbations, which may help clinicians identify subjects at risk. Compared to similar subjects using ICS ± LABA, those taking tiotropium showed a trend towards reduced exacerbation risk, especially in subjects without asthma.

**Trial registration:**

ClinicalTrials.gov NCT00608764, first received 1/28/2008.

**Electronic supplementary material:**

The online version of this article (doi:10.1186/s12890-016-0191-7) contains supplementary material, which is available to authorized users.

## Background

The frequency of exacerbations defines a distinct phenotype of COPD and has implications for prognosis and possibilities for intervention [1]. COPD exacerbations impact the morbidity, mortality, and clinical course of COPD [2–7]. Previous studies examined the association of COPD severity and the frequency of acute exacerbations in an effort to accurately characterize this subset of patients [1]. Additionally, chest computed tomography (CT) features such as bronchial wall thickness [8], emphysema percentage [8], and ratio of pulmonary artery diameter to aortic diameter [9] identify patients susceptible to exacerbations. These studies did not consider differences in medication use and the relationship to exacerbation frequency.

Some commonly prescribed medications have been shown to decrease the rate of exacerbations in randomized clinical trials, including tiotropium (TIO) [10] and long-acting beta-agonists/inhaled corticosteroid (ICS ± LABA) combinations [11]. The relative merits of these two therapies have been compared through a meta-analysis [12] as well as a randomized trial [13]. Although these studies have provided evidence regarding COPD treatment, there still remains a subset of patients who experience frequent exacerbations despite appropriate therapy. It is unclear what factors separate medication non-responders from those who respond more favorably to inhaled medications such as short-acting bronchodilators (SAB), ICS ± LABA, long-acting muscarinic antagonists, or combinations of these medications.

The Genetic Epidemiology of COPD Study (COPDGene, clinicaltrials.gov identifier NCT00608764) is a large observational study of smokers with and without COPD [14]. We examined the differences between COPD subjects with and without exacerbations stratified by common medication use patterns using four separate logistic regression models, analyzing risk factors within each medication usage group to minimize confounding by indication. The primary hypothesis was that unique risk factor profiles for the exacerbator phenotype would exist for subjects on different inhaled medications. In a separate head-to-head analysis, we examined the effect of tiotropium vs. long-acting beta-agonist/inhaled corticosteroids on exacerbation risk.

## Methods

COPDGene is an observational study of COPD conducted at 21 clinical centers in the U.S. (see Additional file 1: Supplementary Data and Methods) designed to discover epidemiologic and genetic risk factors for COPD [8, 14, 15]. It consists of 10,192 current and former Non-Hispanic white or African-American smokers with and without COPD. Each subject underwent spirometry, inspiratory and expiratory chest CT scans, and questionnaires related to demographics, medical history, symptoms and quality of life, in addition to blood samples for genetic analysis. The Longitudinal Follow-Up (LFU) program is a prospective follow-up program [16] in COPDGene using automated telephone and web-based surveys every 3–6 months to monitor incident exacerbations, comorbidities, and death [15]. The LFU dataset (version Oct 12, 2013) used for this investigation included 8465 subjects with a mean follow-up time of 3.35 years. An exacerbation was defined as an episode of increased cough, phlegm, or shortness of breath that lasted >48 h and required treatment with antibiotics, systemic steroids, or both. Severe exacerbations required an emergency room visit or hospitalization. Asthma was defined by self-report of a doctor’s diagnosis of asthma before age 40, as previously reported [17]. The COPDGene Study was approved by Partners Healthcare institutional review board (Protocol # 2007P000554) and institutional review boards at all study sites, and written informed consent was obtained from all subjects. This study was conducted in accordance with the amended Declaration of Helsinki. The St. George's Respiratory Questionnaire was used with permission.

**Fig. 1:**
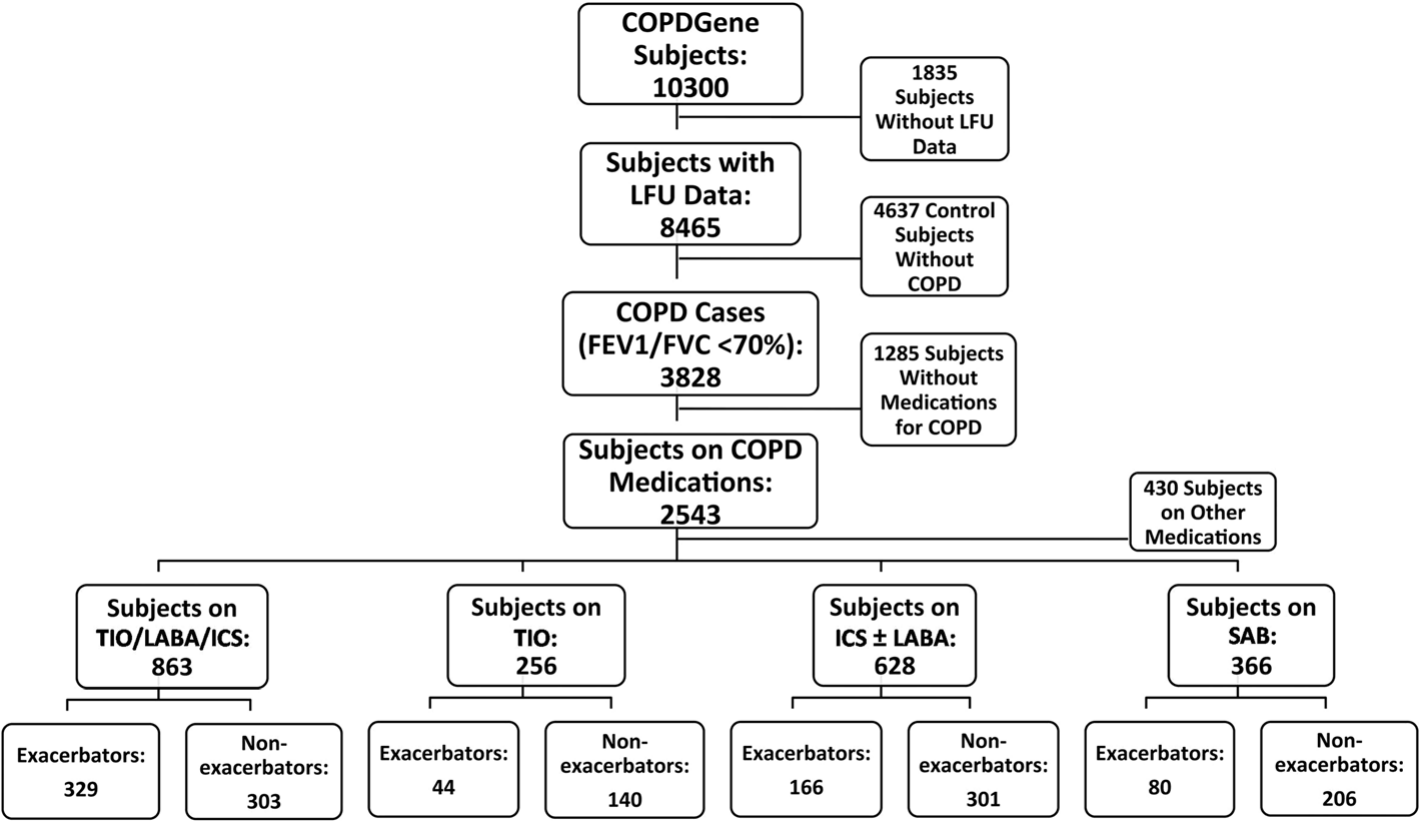
Study subjects. The COPDGene cohort was narrowed to those with LFU data available, then narrowed to subjects with a diagnosis of COPD by GOLD criteria, then to those taking medications. These subjects were divided into four exclusive groups based on medication use, as defined in the Methods section. Exacerbators had one or more exacerbations per year, non-exacerbators had zero exacerbations per year, subjects with between 0 and 1 exacerbations per year were not classified. LFU = Longitudinal follow-up; FEV1/FVC = ratio of forced expiratory volume in one second to forced vital capacity

This analysis included subjects with COPD defined by a forced expiratory volume in one second (FEV1) to forced vital capacity (FVC) ratio <0.7 (GOLD stages 1–4) [18]. Subjects without LFU data and subjects not using inhaled medications were excluded, leaving 2543 subjects. Four medication usage patterns (Fig. 1) were defined at the baseline study visit: 1) Triple therapy with long-acting beta-agonist/inhaled corticosteroid and tiotropium (TIO/LABA/ICS, *N* = 863); 2) tiotropium alone (TIO, *N* = 256); 3) long-acting beta-agonists/inhaled corticosteroid or inhaled corticosteroid alone (ICS ± LABA, *N* = 628); 4) short-acting bronchodilator medications only (SAB, *N* = 366), including albuterol or ipratropium alone or in combination.

**Fig. 2:**
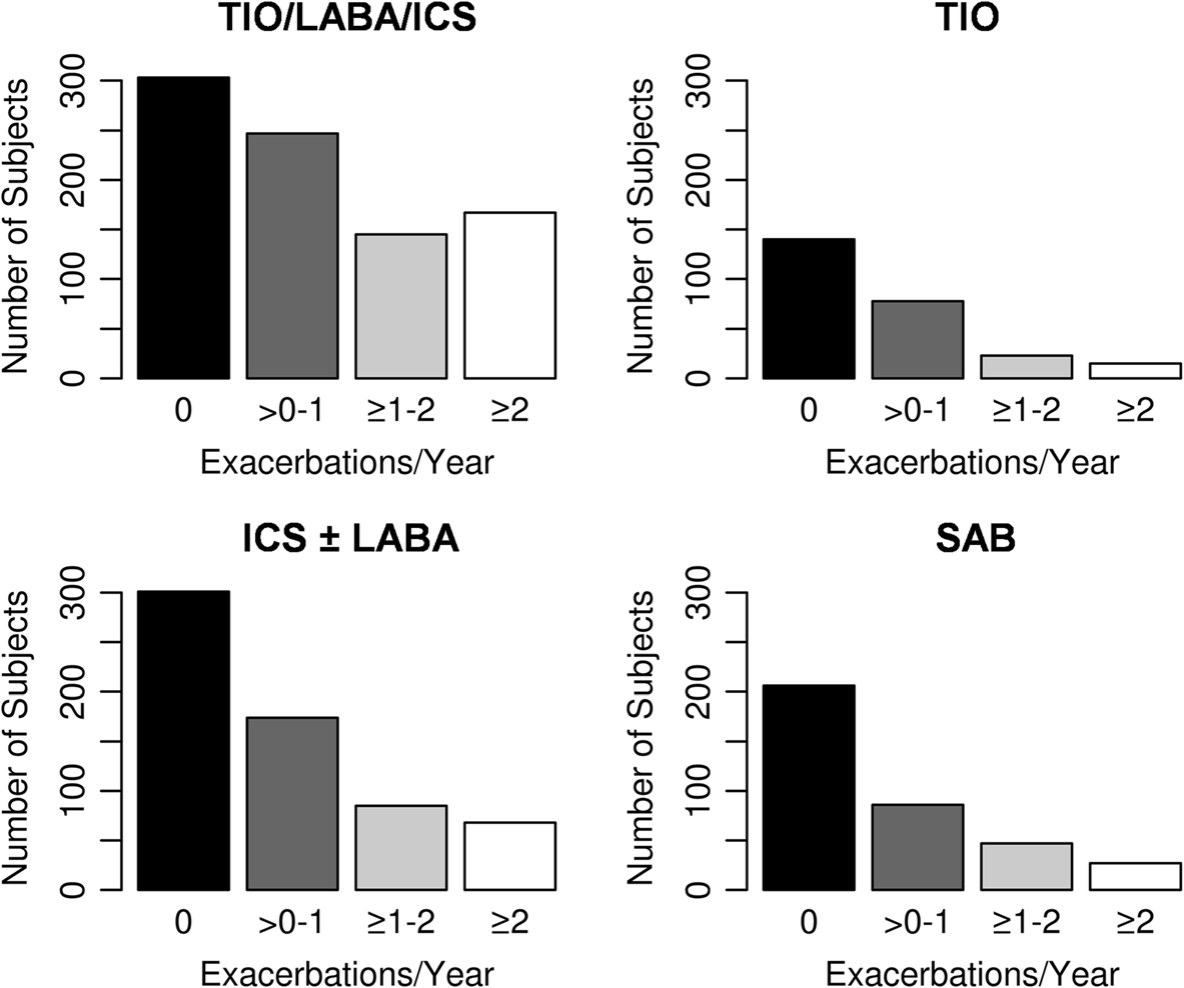
Frequency of COPD exacerbations within the four medication groups. Medication groups are mutually exclusive. TIO/LABA/ICS indicates triple therapy with tiotropium, long-acting beta-agonist/inhaled corticosteroid, TIO indicates tiotropium alone, ICS ± LABA indicates long-acting beta-agonist/inhaled corticosteroid combination therapy, and SAB indicates short-acting bronchodilators alone

Subjects with an exacerbation rate of one or more per year in the LFU were considered exacerbators, those with no exacerbations were considered non-exacerbators, and those with between zero and one exacerbation per year were not classified. The previously described threshold of greater than two exacerbations per year [1] resulted in an insufficient sample size for the subgroup analyses (Fig. 2). Statistical analysis was performed using R software [19].

Additional methods are available in the online supplement (see Additional file 1: Supplementary Data and Methods).

### Within medication group analysis

We compared exacerbators versus non-exacerbators within each medication group for differences in imaging characteristics, symptoms, comorbidities, pulmonary function tests, and demographics. Univariate analysis of binary variables was performed using chi-square statistics and continuous variables using Student's T-test. Significant prospective univariate risk factors were used to build multivariable logistic regression models including a group of common covariates (age, race, gender, FEV1 % predicted).

### Head-to-head analysis

We examined the effect of tiotropium vs long-acting beta-agonist/inhaled corticosteroid on exacerbator phenotype in a logistic regression model using a combined subject pool of the TIO and ICS ± LABA medication groups, who were shown to have similar baseline characteristics. Covariates included baseline differences between the groups. The analysis was repeated after stratification for comorbid asthma.

## Results

### Baseline characteristics

The TIO/LABA/ICS group showed a higher percentage of subjects on oxygen therapy, shorter six minute walk test (6MWT) distance, lower FEV1, higher Modified Medical Research Council dyspnea (MMRC) score, higher total SGRQ score, higher BODE (body mass index, airflow obstruction, dyspnea, and exercise capacity) score, and greater percent emphysema and gas trapping compared to the other groups (Table 1), similar to data previously published by Park et al. [20]. In addition, they showed higher rates of exacerbations/year (1.1) and severe exacerbations/year (0.4) in LFU. These features describe a sicker population with worse functional measures, higher symptom scores, and more disease on chest CT scans.

**Table 1:** Baseline characteristics: COPD subjects included in the longitudinal follow-up data, stratified by medication groups

	All COPD subjects	Chronic oral steroids	TIO/LABA/ICS	TIO	ICS ± LABA	SAB
*N* =	3828	174	863	256	628	366
Gender (female)	2091 (52 %)	87 (50 %)	447 (52 %)	120 (47 %)	305 (49 %)	218 (60 %)
Race (African-American)	736 (19 %)	50 (29 %)	155 (18 %)	35 (14 %)	112 (18 %)	92 (25 %)
Oxygen Therapy	948 (36 %)	87 (50 %)	455 (53 %)	71 (28 %)	165 (26 %)	57 (16 %)
Chronic Bronchitis	968 (30 %)	52 (30 %)	242 (28 %)	65 (25 %)	186 (30 %)	128 (35 %)
Doctor's Diagnosis of Asthma	918 (32 %)	71 (41 %)	271 (31 %)	41 (16 %)	247 (39 %)	107 (29 %)
Frequent Cough Symptom	1597 (47 %)	87 (50 %)	369 (43 %)	118 (46 %)	289 (46 %)	204 (56 %)
Frequent Phlegm Symptom	1739 (52 %)	107 (61 %)	420 (49 %)	115 (45 %)	328 (52 %)	209 (57 %)
Hay Fever	1156 (33 %)	69 (40 %)	263 (30 %)	79 (31 %)	235 (37 %)	113 (31 %)
GERD	1142 (34 %)	54 (31 %)	313 (36 %)	85 (33 %)	220 (35 %)	114 (31 %)
Cardiovascular Disease	748 (22 %)	31 (18 %)	197 (23 %)	63 (25 %)	129 (21 %)	83 (23 %)
Age (years)	63.8 ± 8.3	63.7 ± 8.6	64.5 ± 7.7	66.3 ± 8.1	64.6 ± 8.5	62.5 ± 8.6
Pack Years of Smoking	51.7 ± 28	51.5 ± 29	54.1 ± 26	53.5 ± 28	52.8 ± 30	54.3 ± 29
Current Smoking	1486 (39 %)	42 (24 %)	188 (22 %)	88 (34 %)	202 (32 %)	202 (55 %)
6MWT distance (ft)	1250 ± 380	943 ± 400	1068 ± 350	1216 ± 320	1206 ± 390	1212 ± 370
FEV1 percent predicted	57.2 ± 20	39.3 ± 19	41.7 ± 17	54.4 ± 19	53.1 ± 20	56.1 ± 19
FEV1/FVC	0.52 ± 0.13	0.43 ± 0.12	0.43 ± 0.12	0.49 ± 0.11	0.50 ± 0.13	0.53 ± 0.12
GOLD 1	660 (17 %)	6 (3 %)	21 (2 %)	23 (9 %)	60 (10 %)	44 (12 %)
GOLD 2	1634 (43 %)	37 (21 %)	238 (28 %)	120 (47 %)	275 (44 %)	174 (47 %)
GOLD 3	1022 (27 %)	67 (39 %)	364 (42 %)	84 (33 %)	209 (33 %)	116 (32 %)
GOLD 4	512 (13 %)	64 (37 %)	240 (28 %)	29 (11 %)	84 (13 %)	32 (9 %)
MMRC Score	1.9 ± 1.3	3.0 ± 1.1	2.8 ± 1.1	2.0 ± 1.2	2.3 ± 1.3	2.0 ± 1.4
SGRQ Total Score	36.2 ± 19	57.2 ± 17	49.1 ± 18	38.9 ± 17	42.6 ± 20	41.9 ± 20
BODE score	2.5 ± 1.9	4.4 ± 1.9	4.0 ± 1.7	2.6 ± 1.7	2.9 ± 1.9	2.6 ± 1.8
TLC by CT percent predicted	102.3 ± 17	104.5 ± 17	106.1 ± 18	101.9 ± 15	104.3 ± 17	100.4 ± 16
SRWA-Pi10	3.70 ± 0.14	3.76 ± 0.13	3.72 ± 0.14	3.70 ± 0.14	3.73 ± 0.15	3.74 ± 0.16
Percent Emphysema	12.0 ± 13	18.4 ± 14	19.5 ± 14	12.7 ± 10	12.7 ± 12	8.8 ± 9.4
Percent Gas Trapping	36.4 ± 20	47.7 ± 20	48.7 ± 19	39.0 ± 17	39.3 ± 20	33.8 ± 20
BMI (kg/m^2)	28.0 ± 6.4	27.9 ± 6.3	27.8 ± 6.3	27.8 ± 6.1	28.4 ± 6.5	28.8 ± 7.1
Exacerbations/Year in Follow-Up	0.7 ± 1.4	1.5 ± 2.0	1.1 ± 1.7	0.5 ± 0.9	0.7 ± 1.1	0.6 ± 1.1
Severe Exacerbations/Year	0.2 ± 0.8	0.6 ± 1.3	0.4 ± 0.9	0.1 ± 0.3	0.2 ± 0.6	0.2 ± 0.6

Mean (SD) or N (%) are shown. See Methods for group definitions. *N* (%) or Mean ± SD are shown

*TIO* Tiotropium, *ICS* ± *LABA* long-acting beta-agonist/inhaled corticosteroid, *SAB* short-acting bronchodilator, *6MWT* six minute walk test distance, *TLC* total lung capacity, *CT* computed tomography, *FEV* forced; expiratory volume in one second, *GERD* gastroesophageal reflux disease, *SRWA-Pi10* square root wall; area of a 10 mm airway, *BMI* body mass index

The ICS ± LABA and TIO groups showed broad similarities in baseline characteristics (Table 2). They did not significantly differ in functional measures such as 6MWT distance and FEV1, rates of comorbidities such as GERD, nor in imaging characteristics such as emphysema and gas trapping. Several of the variables, including MMRC score, SGRQ score, and BODE score, showed small statistically significant differences that may not be clinically significant based on accepted standards. The differences in TLC (101.9 % in TIO vs. 104.3 % in ICS ± LABA) and SRWA-Pi10 (3.70 mm in TIO vs. 3.73 mm in ICS ± LABA) are also small. However, there were differences in the proportion of subjects with asthma (16.0 % in TIO vs. 39.3 % in ICS ± LABA) and the mean exacerbation rates (0.5 per year in TIO vs. 0.7 per year in ICS ± LABA). When subjects with asthma were removed, the differences in exacerbation rates, SRWA-Pi10, and SGRQ were no longer significant (see Additional file 1: Supplementary Data and Methods 1, Table S1 and S2).

**Table 2:** Baseline characteristics: comparison of COPD subjects within the tiotropium or long acting beta-agonists/inhaled corticosteroid groups

	TIO	ICS ± LABA	*p*-value
Gender (female, %)	46.9	48.6	0.7
Race (Non-Hispanic White, %)	86.3	82.2	0.2
Oxygen Therapy (%)	27.7	26.3	0.7
Chronic Bronchitis (%)	25.4	29.6	0.2
Doctor's Diagnosis of Asthma (%)	16.0	39.3	**<0.001**
Frequent Cough Symptom (%)	46.1	46.0	1.0
Frequent Phlegm Symptom (%)	44.9	52.2	0.06
Hay Fever (%)	30.9	37.4	0.2
GERD (%)	33.2	35.0	0.7
Cardiovascular disease (%)	24.6	20.5	0.2
Age (years)	66.3	64.6	**0.006**
Current Smoking	34.4	32.2	0.6
Pack-Years of Smoking	53.5	52.8	0.7
6MWT distance (ft)	1216	1206	0.7
FEV1 percent predicted	54.4	53.1	0.3
FEV1/FVC	0.49	0.50	0.5
MMRC Score	2.0	2.3	**0.006**
SGRQ Total Score	38.9	42.6	**0.007**
BODE score	2.6	2.9	**0.02**
TLC by CT percent predicted	101.9	104.3	**0.04**
SRWA-Pi10	3.70	3.73	**0.003**
Percent Emphysema	12.7	12.7	1.0
Percent Gas Trapping	39.0	39.3	0.8
BMI (kg/m^2)	27.8	28.4	0.2
Exacerbations/Year in Follow-Up	0.5	0.7	**0.004**

Means or percentages are shown. *P*-values < 0.05 are in bold

*6MWT* six minute walk test, *TLC* total lung capacity, *CT* computed tomography, *FEV1* forced expiratory volume in one second, *BD* bronchodilator, *GERD* gastroesophageal reflux disease, *BMI* body mass index

The SAB group contained 366 subjects (Table 1), with the best lung function (FEV1 56.1 % predicted) and one of the lowest exacerbation rates (0.6 per year). Despite this, 148 (40 %) of SAB subjects had GOLD stage 3 or 4 disease. In addition, 80 (22 %) SAB subjects had greater than one exacerbation per year, while 31 (8.5 %) had two or more exacerbations per year. There was a higher proportion of African-Americans than in other groups, and the SAB group had the highest proportion with chronic bronchitis. Functional measurements such as 6MWT were better compared with other groups, and they had the least emphysema.

The 174 subjects on chronic oral steroids tended to have lower lung function (FEV1 39.3 % predicted) and an exacerbation rate (1.6 per year) that was comparable to other groups despite use of chronic systemic steroids. Subjects on chronic oral steroids in COPDGene have been described previously [21].

### Within medication group analysis

In univariate analysis, risk factors for the exacerbator phenotype were discovered within each medication group (see Additional file 1: Supplementary Data and Methods 1, Table S3). Notable risk factors consistent across all medication groups were frequent cough symptoms and higher TLC. GERD, asthma, and higher SGRQ score were statistically significant risk factors of exacerbator status in the TIO/LABA/ICS, TIO, and ICS ± LABA groups, but not in the SAB group. The presence of chronic bronchitis and of daily phlegm symptoms were not significant predictors in subjects taking tiotropium (TIO/LABA/ICS and TIO groups), but were predictors in subjects not taking tiotropium (ICS ± LABA and SAB groups). Similarly, emphysema on CT was predictive of exacerbator phenotype only in subjects not receiving tiotropium. Bronchodilator (BD) responsiveness as measured by absolute change in FEV1 was not a significant risk factor in subjects receiving ICS ± LABA combinations (with or without TIO), but was a significant risk factor in subjects without ICS ± LABA (TIO and SAB groups). Prior venous thromboembolism, obstructive sleep apnea, higher body mass index (BMI), and congestive heart failure were not associated with exacerbator phenotype. Current smoking status was not a risk factor in any medication group, nor was lifetime smoking exposure, measured in pack-years (Additional file 1: Supplementary Data and Methods, Table S3).

Logistic regression was performed within each medication group using the significant univariate risk factors as covariates, in addition to age, race, sex, and FEV1 (Table 3). Risk factors of female gender, higher SGRQ score, prior pneumonia, and GERD remained significant in the TIO/LABA/ICS group, while the presence of co-morbid asthma showed a trend towards significance. Younger age, asthma, greater gas-trapping by CT, and GERD were statistically significant risk factors in the TIO group, while female gender and African-American race approached significance. Female gender, higher SGRQ score, and GERD were risk factors in the ICS ± LABA group, while history of pneumonia, hay fever, and cardiovascular disease all approached statistical significance. In the SAB group, female gender, home oxygen therapy, and frequent cough symptom were all statistically significant risk factors, while higher FEV1 change post-BD approached statistical significance. Statistically significant results are summarized in Table 4.

**Table 3:** Within medication group analysis: logistic regression models for the exacerbator phenotype

	Odds ratio	Lower CI	Upper CI	*p*-value
A. Tiotropium/Inhaled corticosteroid/Long acting beta-agonist				
Age, years	0.98	0.95	1.00	0.1
Female Gender	1.53	1.05	2.21	**0.03**
African-American Race	0.69	0.44	1.09	0.1
FEV1 Percent Predicted	1.00	0.98	1.02	0.9
TLC by CT percent predicted	1.01	0.99	1.02	0.3
SGRQ Total Score	1.02	1.00	1.03	**0.02**
MMRC Score	0.85	0.60	1.21	0.4
BODE Score	1.08	0.80	1.47	0.6
Frequent Cough Symptom	1.11	0.75	1.64	0.6
Doctor's Diagnosis of Asthma	1.19	0.98	1.45	0.08
Hay Fever	1.01	0.81	1.27	0.9
Prior Pneumonia	1.36	1.03	1.80	**0.03**
GERD	1.62	1.11	2.38	**0.01**
B. Tiotropium				
Age, years	0.93	0.87	1.00	**0.04**
Female Gender	2.64	0.95	7.33	0.06
African-American Race	0.15	0.02	1.28	0.08
FEV1 percent predicted	1.02	0.99	1.06	0.1
FEV1 change with bronchodilator (L)	2.99	0.10	87.43	0.5
TLC by CT percent predicted	1.00	0.97	1.04	0.9
Percent Gas Trapping	1.06	1.02	1.11	**0.01**
SGRQ Total Score	1.01	0.98	1.04	0.4
Frequent Cough Symptom	1.69	0.64	4.47	0.3
Doctor's Diagnosis of Asthma	1.57	1.06	2.32	**0.03**
GERD	2.75	1.10	6.88	**0.03**
C. Long acting beta-agonist/inhaled corticosteroid				
Age, years	1.00	0.96	1.03	0.8
Female Gender	1.90	1.19	3.05	**0.01**
African-American Race	0.71	0.39	1.29	0.3
FEV1 percent predicted	0.98	0.96	1.01	0.1
Resting Oxygen Saturation (%)	0.97	0.90	1.04	0.4
6MWT distance	1.00	1.00	1.00	0.5
FEV1 change with bronchodilator (L)	1.65	0.42	6.39	0.5
TLC by CT percent predicted	1.01	0.99	1.02	0.3
Percent Emphysema	1.02	0.99	1.04	0.2
MMRC Score	1.04	0.71	1.54	0.8
SGRQ Total Score	1.03	1.01	1.04	**0.01**
BODE Score	0.96	0.68	1.35	0.8
Frequent Cough Symptom	1.50	0.91	2.45	0.1
Doctor's Diagnosis of Asthma	1.00	0.77	1.29	1.0
Hay Fever	1.27	0.98	1.66	0.07
Prior pneumonia	1.35	0.99	1.84	0.06
GERD	1.96	1.21	3.15	**0.01**
Cardiovascular disease	1.68	0.96	2.91	0.07
D. Short acting bronchodilators				
Age, years	1.01	0.97	1.05	0.8
Female Gender	2.56	1.30	5.03	**0.01**
African-American Race	0.53	0.23	1.21	0.1
FEV1 percent predicted	0.98	0.96	1.01	0.1
Oxygen Therapy	2.47	1.11	5.51	**0.03**
FEV1 change with bronchodilator (L)	5.60	0.97	32.41	0.05
TLC by CT percent predicted	1.00	0.98	1.03	0.7
Percent Emphysema	1.03	0.99	1.08	0.2
BODE Score	1.00	0.78	1.28	1.0
Frequent Cough Symptom	2.14	1.13	4.06	**0.02**
Hay Fever	1.19	0.86	1.64	0.3
Prior Pneumonia	1.08	0.75	1.56	0.7

*P*-values <0.05 are in bold

**Table 4:** Within medication group analysis: summary of exacerbation risk factors by medication group

	TIO/LABA/ICS	TIO	ICS ± LABA	SAB
Younger Age (years)		+		
Doctor's Diagnosis of Asthma	/	+		
Frequent Cough Symptom				+
Female Gender	+	/	+	+
GERD	+	+	+	
Oxygen Therapy				+
Higher Percent Gas Trapping		+		
Prior Pneumonia	+		/	
Higher SGRQ Total Score	+		+	

(+) indicates that the variable was a statistically significant predictor of exacerbator status in the corresponding medication group (see Table 3). (/) indicates that the variable approached statistical significance (*p* < 0.1). Direction of association is noted in the variable text

### Head-to-head analysis

In the analysis of each group’s baseline characteristics, we determined that the TIO and ICS ± LABA groups represented broadly similar populations of subjects in terms of severity, imaging characteristics, and symptoms. These similarities allowed us to combine these groups to analyze exacerbation risk factors in a head-to-head fashion while limiting confounding by indication. We compared the effects of tiotropium vs. ICS ± LABA therapy on prospective exacerbations in a logistic regression model combining subjects in the TIO and ICS ± LABA groups (Table 5) adjusted for the baseline differences between the groups, including age, asthma, MMRC score, SGRQ score, BODE score, and TLC. There was a trend towards a reduction in exacerbation risk for tiotropium (OR 0.69, *p* = 0.09). The logistic regression models were repeated after stratifying by asthma status. In subjects without a history of comorbid asthma, there was a stronger trend toward reduction in exacerbation risk from tiotropium (OR 0.56, *p* = 0.05). In only the subjects with comorbid asthma, this association disappeared (OR 1.03, *p* = 1.0).

**Table 5:** Head-to-head analysis: logistic regression models for the effect of tiotropium vs. long-acting beta-agonist/inhaled corticosteroid on exacerbator status

	Odds ratio	Lower CI	Upper CI	*p*-value
A. All subjects using either TIO or ICS ± LABA (*N* = 602)				
Tiotropium Usage	0.69	0.45	1.06	0.09
Doctor's Diagnosis of Asthma	1.22	1.01	1.47	0.04
Age (years)	1.00	0.98	1.02	1.0
MMRC Score	0.97	0.75	1.25	0.8
SGRQ Total Score	1.03	1.01	1.04	<0.001
BODE Score	1.08	0.92	1.26	0.4
TLC by CT percent predicted	1.02	1.00	1.03	0.01
B. Excluding subjects with doctor diagnosed asthma (*N* = 341)				
Tiotropium Usage	0.56	0.31	1.00	0.05
Age (years)	1.00	0.96	1.03	0.9
MMRC Score	1.03	0.70	1.50	0.9
SGRQ Total Score	1.04	1.01	1.06	<0.001
BODE Score	0.96	0.77	1.21	0.8
TLC by CT percent predicted	1.02	1.00	1.04	0.02
C. Only subjects with doctor diagnosed asthma (*N* = 198)				
Tiotropium Usage	1.03	0.39	2.70	1.0
Age (years)	1.01	0.97	1.05	0.6
MMRC Score	0.89	0.59	1.35	0.6
SGRQ Total Score	1.02	1.00	1.04	0.06
BODE Score	1.21	0.94	1.57	0.1
TLC by CT percent predicted	1.01	0.99	1.03	0.2

## Discussion

Using prospective observational data from the COPDGene Study, we identified risk factors for exacerbations within four groups of subjects with common medication use patterns. While comparison across medication groups is confounded by indication, we found substantial similarities between the subjects using ICS ± LABA and those using TIO. Controlling for baseline differences in a head-to-head analysis, we saw a trend towards an association with reduced exacerbation risk for tiotropium compared to ICS ± LABA, especially in subjects without the diagnosis of asthma. We also demonstrated the feasibility of using self-reported medication data from an observational study to generate hypotheses regarding drug treatment effects.

The interplay of comorbid conditions with COPD exacerbations has been previously described [22, 23], and the influence of comorbidities on COPD exacerbations is also shown in our study. Respiratory comorbidities such as asthma and prior pneumonia were risk factors in some groups. GERD has previously been described as a risk factor for more frequent COPD exacerbations [24] and our data showed a similar effect in all medication groups except for those using only short-acting agents. Notably, other cardiopulmonary comorbidities such as prior venous thromboembolism, obstructive sleep apnea, higher BMI, and congestive heart failure did not show an association. Previous studies have shown an association between ongoing smoking and exacerbation risk [25–27]. However, current smoking and cumulative pack-year exposure were not risk factors in any group within our study, despite the acute [28] and chronic inflammatory effects of cigarette smoke on airways [29] and the immune system [30].

The baseline characteristics of the TIO and ICS ± LABA groups were similar across many clinical parameters. Although SGRQ and MMRC scores were statistically different between the two groups, these did not exceed thresholds for minimal clinically important differences [31, 32]. The similarities between these baseline characteristics make possible comparison between the two groups with less concern of confounding by indication. The similarity between the two groups was more clearly seen among the non-asthmatic subjects. These findings reflect current clinical practice guidelines, in which administration of a long-acting beta-agonist/inhaled corticosteroid or long-acting muscarinic-antagonist are both recommended for patients with FEV1 < 60 % predicted and stable symptoms [33]. The overall rate of exacerbations among this population was lower than previously published randomized controlled trials data of TIO [3, 13] or ICS ± LABA [13], however those trials enrolled patients with more severe airflow obstruction.

The number of subjects with asthma in the ICS ± LABA group was higher than in the TIO group. It is plausible that concurrent asthma may lead a provider to prescribe ICS ± LABA combinations [34] given the prominent role of inhaled corticosteroids as anti-inflammatory therapy in asthma [35]. Alternatively, subjects with asthma and COPD may have had greater symptomatic response to ICS ± LABA, creating a preference for this medication among asthma-COPD overlap patients. In previous COPDGene publications, the asthma-COPD overlap syndrome has been shown to be a relevant clinical entity, associated with worse health-related quality of life and more frequent exacerbations [17, 36]. These findings were recapitulated in our analysis, with subjects with asthma-COPD overlap taking either TIO or ICS ± LABA showing a higher rate of exacerbations than their non-asthmatic counterparts, as well as a trend towards higher SGRQ scores.

In this observational study, we observed a trend towards an association with reduction in exacerbation risk in subjects taking tiotropium compared to ICS ± LABA. The trend was stronger in subjects without asthma. Conflicting randomized trial literature exists on the choice of tiotropium or long-acting beta-agonist/inhaled corticosteroid in advanced stage COPD. The INSPIRE trial [13] showed no difference in rates of exacerbations among COPD subjects randomized to ICS ± LABA vs. TIO, and subjects were excluded from INSPIRE if they had significant bronchodilator response or asthma [37]. However INSPIRE subjects had more severe airflow obstruction (mean FEV1 39.3 % predicted vs. 48.3 % predicted in our study), so their comparison of TIO and ICS/LABA may not generalize to COPDGene. Conversely, a recent meta-analysis of randomized controlled trials comparing tiotropium vs. long-acting beta-agonist (with 35–56 % of patients taking inhaled corticosteroid as well) in stable COPD showed a reduction in exacerbations with tiotropium.

Previous literature comparing COPD medication regimens with and without inhaled corticosteroids have largely focused on comparisons of LABA alone versus ICS + LABA, without comparisons of long-acting muscarinics versus an ICS ± LABA regimen. A randomized trial conducted in Canada in subjects with moderate to severe COPD showed a decreased exacerbation rate in subjects receiving fluticasone as part of a triple therapy regimen of tiotropium/fluticasone/salmeterol compared with those receiving tiotropium alone or tiotropium/salmeterol [38]. The TORCH trial [11] showed a decreased annual exacerbation rate in subjects with moderate to severe COPD receiving fluticasone/salmeterol therapy compared with those receiving salmeterol alone. More recently, the WISDOM trial [39] demonstrated that the time to first moderate or severe COPD exacerbation was similar in subjects with severe and very severe COPD continuing triple therapy with tiotropium/salmeterol/fluticasone or undergoing controlled withdrawal of inhaled corticosteroid therapy, which cast doubt on the added benefit of corticosteroids for those with stable COPD. Finally, the longitudinal cohort study by Gershon et al. from 2014 [40] reviewed database information in elderly COPD subjects without asthma starting LABA or ICS ± LABA therapy and showed that those subjects using LAMAs did not have a better outcome on the composite outcome of hospitalization or death. Given the conflicting evidence regarding the optimal choice of inhaled therapy for patients with moderate to severe COPD, we contend that our data adds to the debate questioning the added benefit of inhaled corticosteroids in these patients, and is hypothesis-generating for additional investigation comparing ICS ± LABA versus tiotropium alone.

Our study has several limitations. The medication usage data is based upon subject report at the initial study visit, and was not updated during the longitudinal follow-up. Because of the lack of follow-up medication data, we are unable to account for medication changes that might influence inclusion and analysis of a subject in a particular medication group. Self-report of exacerbations could introduce bias, however this self-reported definition has been used in previous work in COPDGene as well as other COPD studies such as ECLIPSE [1]. LFU calls were completed every 3 to 6 months, so some exacerbation events may have been missed. Therefore we dichotomized subjects into exacerbators and non-exacerbators, rather than analyzing exacerbation counts. Our definition of exacerbators used a threshold of ≥1 exacerbation per year, which differs from previous studies which used ≥2 exacerbations per year [1] and may limit direct comparison of results. COPDGene subjects had a lower overall rate of exacerbations than other study populations. Using a threshold of ≥2 exacerbations per year led to small sample sizes in the medication groups (see Fig. 2) that would have limited our power to detect associations. The definition of asthma in this study was based on self-reported diagnosis by a clinician, which has been previously used in COPDGene [17]. The diagnosis of asthma may have influenced a prescriber’s choice of inhaled corticosteroids as a controller medication. In order to minimize confounding by indication, we compared exacerbation risk factors within subjects reporting the same medication usage pattern. The characteristics of subjects using TIO and ICS ± LABA were similar, so we were able to compare these medications in a prospective observational design. Our results suggest that tiotropium may be associated with decreased exacerbations compared with ICS ± LABA among patients without concurrent asthma. However a randomized clinical trial would be needed to confirm this hypothesis.

## Conclusions

In summary, this study examined risk factors for future exacerbations in COPD subjects using common medications, which may help providers identify individuals at higher risk of exacerbations and influence treatment strategies or discussions of action plans. We confirmed previous associations of GERD and female gender with exacerbation risk in certain subsets, and found that previous pneumonia, higher SGRQ scores, and gas trapping on CT scans were risk factors for exacerbations in subjects using certain medications. The head-to-head analysis also showed a trend towards a reduced exacerbation risk with tiotropium compared to ICS ± LABA in COPD subjects without concurrent asthma, which could be viewed as hypothesis-generating for a clinical trial. This study may also add to the body of literature questioning the added benefit of ICS in moderate to severe COPD.

### Ethics approval and consent to participate

The COPDGene Study was approved by Partners Healthcare institutional review board (Protocol # 2007P000554) and institutional review boards at all study sites, and written informed consent was obtained from all subjects.

### Consent for publication

Not Applicable

### Availability of data and materials

COPDGene data is available on dbGAP (http://www.ncbi.nlm.nih.gov/gap, accession number phs000179.v4.p2)

## Additional file

Additional file 1: Supplementary Data and Methods. (DOCX 38 kb)

## Abbreviations

6MWT: six minute walk test
BD: bronchodilator
BMI: body mass index
BODE: body mass index, airflow obstruction, dyspnea, and exercise capacity score
COPD: chronic obstructive pulmonary disease
COPDGene: The Genetic Epidemiology of COPD Study
CT: computed tomography
FEV1: forced expiratory volume in one second
FVC: forced vital capacity
GERD: gastroesophageal reflux disease
GOLD: Global Initiative for Obstructive Lung Disease
ICS ± LABA: long-acting beta-agonist/inhaled corticosteroid
LFU: COPDGene biannual telephone- and web-based longitudinal follow-up program
MMRC: Modified Medical Research Council Scale for Dyspnea
OR: odds ratio
SAB: short-acting bronchodilator
SGRQ: St. George's Respiratory Questionnaire
SRWA-Pi10: square root of wall area of a hypothetical 10 mm internal perimeter airway
TIO: tiotropium
TLC: total lung capacity
