# Supplementary material for: Risk factors for COPD exacerbations in inhaled medication users: the COPDGene study biannual longitudinal follow-up prospective cohort
Source: BMC Pulm Med. 2016 Feb 10;16:28. doi: 10.1186/s12890-016-0191-7 (PMC4748594; doi:10.1186/s12890-016-0191-7)
Supplement: Additional file 1: — Supplementary Data and Methods. (DOCX 38 kb) [file 12890_2016_191_MOESM1_ESM.docx]

Supplementary Methods

COPDGene was conducted at 21 clinical centers: Ann Arbor VA, Ann Arbor, MI; Baylor College of Medicine, Houston, TX; Brigham and Women’s Hospital, Boston, MA; Columbia University, New York, NY; Duke University Medical Center, Durham, NC; Health Partners Research Foundation, Minneapolis, MN; Johns Hopkins University, Baltimore, MD; Los Angeles Biomedical Research Institute at Harbor UCLA Medical Center, Los Angeles, CA; Michael E. DeBakey VAMC, Houston, TX; Minneapolis VA, Minneapolis, MN; Morehouse School of Medicine, Atlanta, GA; National Jewish Health, Denver, CO; Reliant Medical Group, Worcester, MA; Temple University, Philadelphia, PA; University of Alabama, Birmingham, AL; University of California, San Diego, CA; University of Iowa, Iowa City, IA; University of Michigan, Ann Arbor, MI; University of Minnesota, Minneapolis, MN; University of Pittsburgh, Pittsburgh, PA; University of Texas Health Science Center at San Antonio, San Antonio, TX.

This analysis included subjects with COPD defined by a forced expiratory volume in one second (FEV1) to forced vital capacity (FVC) ratio <0.7 (GOLD stages 1-4)([1](#_ENREF_1)). Subjects without LFU data and subjects not using inhaled medications were excluded, leaving 2543 subjects. Four medication usage patterns (Figure 1) were defined at the baseline study visit: 1) Triple therapy with long-acting beta-agonist/inhaled corticosteroid and tiotropium (TIO/LABA/ICS, N = 863); 2) tiotropium alone (TIO, N = 256); 3) long-acting beta-agonists/inhaled corticosteroid or inhaled corticosteroid alone (LABA/ICS, N = 628); 4) short-acting bronchodilator medications only (SAB, N = 366), including albuterol or ipratropium alone or in combination. Short-acting bronchodilators and chronic theophylline (n=143) were allowed in all groups. Subjects on chronic oral corticosteroids (N = 174), other combinations of COPD medications (N=60), or with incomplete medication data (N=196) were excluded.

Asthma was defined by self-report of a doctor’s diagnosis of asthma before age 40, as previously reported([2](#_ENREF_2)). Cardiovascular disease included self-report of coronary artery disease, myocardial infarction, angioplasty, coronary artery bypass grafting, angina, congestive heart failure, and peripheral vascular disease([3](#_ENREF_3)). For small airway assessment, the square root of the wall area of a theoretical airway with 10mm internal perimeter (SRWA-Pi10) method was used([4](#_ENREF_4)). Total lung capacity (TLC) was measured by inspiratory CT scan. Emphysema was measured as the percent of lung voxels on inspiratory chest CT scan with attenuation of less than -950 Hounsfield Units, using SLICER software([5-7](#_ENREF_5)). Gas trapping was measured as the percent of lung voxels on expiratory chest CT scan with attenuation of less than -856 Hounsfield Units.

References

1. Pauwels RA, Buist AS, Calverley PM, Jenkins CR, Hurd SS. Global strategy for the diagnosis, management, and prevention of chronic obstructive pulmonary disease. NHLBI/WHO Global Initiative for Chronic Obstructive Lung Disease (GOLD) Workshop summary. *Am J Respir Crit Care Med* 2001; 163: 1256-1276.

2. Hardin M, Silverman EK, Barr RG, Hansel NN, Schroeder JD, Make BJ, Crapo JD, Hersh CP. The clinical features of the overlap between COPD and asthma. *Respir Res* 2011; 12: 127.

3. Black-Shinn JL, Kinney GL, Wise AL, Regan EA, Make B, Krantz MJ, Barr RG, Murphy JR, Lynch D, Silverman EK, Crapo JD, Hokanson JE. Cardiovascular Disease is Associated with COPD Severity and Reduced Functional Status and Quality of Life. *Copd* 2014; 11: 546-551.

4. Nakano Y, Wong JC, de Jong PA, Buzatu L, Nagao T, Coxson HO, Elliott WM, Hogg JC, Pare PD. The prediction of small airway dimensions using computed tomography. *Am J Respir Crit Care Med* 2005; 171: 142-146.

5. Regan EA, Hokanson JE, Murphy JR, Make B, Lynch DA, Beaty TH, Curran-Everett D, Silverman EK, Crapo JD. Genetic epidemiology of COPD (COPDGene) study design. *Copd* 2010; 7: 32-43.

6. Hersh CP, Washko GR, Jacobson FL, Gill R, Estepar RS, Reilly JJ, Silverman EK. Interobserver variability in the determination of upper lobe-predominant emphysema. *Chest* 2007; 131: 424-431.

7. Hersh CP, Washko GR, Estepar RS, Lutz S, Friedman PJ, Han MK, Hokanson JE, Judy PF, Lynch DA, Make BJ, Marchetti N, Newell JD, Jr., Sciurba FC, Crapo JD, Silverman EK. Paired inspiratory-expiratory chest CT scans to assess for small airways disease in COPD. *Respir Res* 2013; 14: 42.

**Table S1.** Characteristics of subjects using tiotropium compared to subjects using inhaled corticosteroids/long-acting beta-agonists (LABA/ICS) after removal of subjects with doctor-diagnosed asthma

|  | **TIO** | **LABA/ICS** | **p-Value** |
| --- | --- | --- | --- |
| **Gender (female,%)** | 46.7 | 54.8 | 0.1 |
| **Race (Non-Hispanic White,%)** | 87.5 | 86.2 | 0.8 |
| **Oxygen Therapy (%)** | 27.2 | 29.8 | 0.6 |
| **Chronic Bronchitis (%)** | 25.5 | 27.6 | 0.7 |
| **Frequent Cough Symptom (%)** | 44.6 | 41.4 | 0.5 |
| **Frequent Phlegm Symptom (%)** | 45.7 | 51.0 | 0.3 |
| **Hay Fever (%)** | 26.1 | 26.9 | 1.0 |
| **GERD (%)** | 29.9 | 30.5 | 1.0 |
| **Cardiovascular disease (%)** | 23.4 | 20.2 | 0.5 |
| **Age (years)** | 66.8 | 65.9 | 0.2 |
| **Pack-Years of Smoking** | 53.8 | 57.0 | 0.3 |
| **6MWT distance (ft)** | 1205 | 1178 | 0.4 |
| **FEV1 percent predicted** | 53.7 | 52.1 | 0.4 |
| **FEV1/FVC** | 0.49 | 0.49 | 0.8 |
| **MMRC Score** | 1.9 | 2.33 | **0.001** |
| **SGRQ Score** | 38.6 | 41.4 | 0.09 |
| **BODE score** | 2.6 | 3.0 | **0.02** |
| **TLC percent predicted by CT** | 101.5 | 105.4 | **0.01** |
| **SRWA-Pi10** | 3.70 | 3.71 | 0.4 |
| **Percent Emphysema** | 13.4 | 14.8 | 0.2 |
| **Percent Gas Trapping** | 40.0 | 41.8 | 0.3 |
| **BMI (kg/m^2)** | 27.3 | 27.3 | 0.9 |
| **Exacerbations/Year in Follow-Up** | 0.4 | 0.6 | 0.06 |
| Means or percentages are shown. P-values<0.05 are in bold. 6MWT = Six minute walk test; TLC = Total lung capacity; CT = Computed tomography; FEV1 = Forced expiratory volume in one second; BD = Bronchodilator; GERD = Gastroesophageal reflux disease; BMI = Body Mass Index; | | | |

**Table S2.** Characteristics of subjects using tiotropium compared to subjects using inhaled corticosteroids/long-acting beta-agonists (LABA/ICS) among only those subjects with doctor-diagnosed asthma

|  | **TIO** | **LABA/ICS** | **p-Value** |
| --- | --- | --- | --- |
| **Gender (female,%)** | 48.8 | 38.8 | 0.30 |
| **Race (Non-Hispanic White,%)** | 80.5 | 76.1 | 0.68 |
| **Oxygen Therapy (%)** | 29.3 | 19.4 | 0.22 |
| **Chronic Bronchitis (%)** | 24.4 | 30.4 | 0.55 |
| **Frequent Cough Symptom (%)** | 48.8 | 51.8 | 0.85 |
| **Frequent Phlegm Symptom (%)** | 46.3 | 53.4 | 0.50 |
| **Hay Fever (%)** | 46.3 | 51.4 | 1.00 |
| **GERD (%)** | 41.5 | 39.3 | 0.93 |
| **Cardiovascular disease (%)** | 36.6 | 22.3 | 0.07 |
| **Age (years)** | 65.1 | 63.1 | 0.14 |
| **Pack-Years of Smoking** | 52.6 | 47.6 | 0.21 |
| **6MWT distance (ft)** | 1309 | 1240 | 0.27 |
| **FEV1 percent predicted** | 58.4 | 55.5 | 0.41 |
| **FEV1/FVC** | 0.51 | 0.52 | 0.68 |
| **MMRC Score** | 2.2 | 2.1 | 0.71 |
| **SGRQ Score** | 38.6 | 43.0 | 0.21 |
| **BODE score** | 2.4 | 2.7 | 0.36 |
| **TLC percent predicted by CT** | 102.1 | 102.7 | 0.83 |
| **SRWA-Pi10** | 3.69 | 3.74 | **0.04** |
| **Percent Emphysema** | 10.1 | 9.9 | 0.92 |
| **Percent Gas Trapping** | 34.0 | 35.2 | 0.71 |
| **BMI (kg/m^2)** | 28.9 | 29.4 | 0.63 |
| **Exacerbations/Year in Follow-Up** | 0.7 | 0.9 | 0.25 |

| Means or percentages are shown. P-values<0.05 are in bold. 6MWT = Six minute walk test; TLC = Total lung capacity; CT = Computed tomography; FEV1 = Forced expiratory volume in one second; BD = Bronchodilator; GERD = Gastroesophageal reflux disease; BMI = Body Mass Index; |
| --- |

**Table S3:** Univariate exacerbation risk factors within each medication group

|  | **TIO/LABA/ICS** | **TIO** | **LABA/ICS** | **SAB** |
| --- | --- | --- | --- | --- |
| **Younger Age** | **+** | **+** |  |  |
| **African American Race** |  |  |  | **+** |
| **Female Gender** | **+** |  |  |  |
| **Current Smoking** |  |  |  |  |
| **Pack-Years of smoking** |  |  |  |  |
| **Lower Resting Oxygen Saturation (%)** |  |  | **+** |  |
| **Shorter 6MWT Distance (ft)** |  |  | **+** |  |
| **Oxygen Therapy** |  |  | **+** | **+** |
| **Lower FEV1 percent predicted (%)** |  |  | **+** | **+** |
| **Lower FVC percent predicted (%)** |  |  | **+** |  |
| **Lower FEV1/FVC** |  |  | **+** | **+** |
| **Positive BD Response, ATS/ERS definition** |  | **+** |  |  |
| **Higher %Change in FEV1 post BD** |  |  |  | **+** |
| **Higher Change in FEV1 post BD (ml)** |  | **+** |  | **+** |
| **Higher TLC percent predicted by CT** | **+** | **+** | **+** | **+** |
| **Higher Percent Total Emphysema** |  |  | **+** | **+** |
| **Higher Percent Gas Trapping** |  | **+** | **+** | **+** |
| **Lower SRWA-Pi10 Measure** |  |  |  |  |
| **Higher MMRC Score** | **+** |  | **+** |  |
| **Higher SGRQ Total Score** | **+** | **+** | **+** |  |
| **Higher BODE Score** | **+** |  | **+** | **+** |
| **Frequent Cough Symptom** | **+** | **+** | **+** | **+** |
| **Frequent Phlegm Symptom** |  |  | **+** | **+** |
| **Chronic Bronchitis** |  |  | **+** | **+** |
| **Doctor's Diagnosis of Asthma** | **+** | **+** | **+** |  |
| **Hay Fever** | **+** |  | **+** | **+** |
| **GERD** | **+** | **+** | **+** |  |
| **Cardiovascular Disease** |  |  | **+** |  |
| **Higher BMI (kg/m^2)** |  |  |  |  |
| (**+**) indicates that the variable was a statistically significant univariate predictor of exacerbator status in the corresponding medication group using Student’s t-test or chi-square test as appropriate. Direction of association is noted in the variable text. TIO = Tiotropium, LABA/ICS = Long-acting Beta-agonist, Inhaled Corticosteroid; SAB = Short acting, Bronchodilator; 6MWT = Six minute walk test; TLC = Total lung capacity; CT = Computed tomography; FEV1 = Forced expiratory volume in one second; BD = Bronchodilator; GERD = Gastroesophageal reflux; SRWA-Pi10 = Square Root Wall Area of a 10mm airway; | | | | |
